# Estimation of biological aging based on T-cell differentiation trajectories: emerging and future avenues

**DOI:** 10.3389/fragi.2025.1684051

**Published:** 2025-11-20

**Authors:** Aarav M. Bhasin, Ishaan K. Marwaha, Lahiri S. Nooka

**Affiliations:** 1 Johns Creek High School, Johns Creek, GA, United States; 2 Pope High School, Marietta, GA, United States; 3 The Westminster Schools, Buckhead, GA, United States

**Keywords:** T-cell aging, immunosenescence, aging clock, T-cell dynamics, immune aging, aging biomarker, biological aging, aging and cancer

## Abstract

The relationship between disease onset and chronological age varies between individuals, driving the need for a more accurate, universal biomarker of biological aging. Among the emerging alternatives, the immune system represents a universally shared, complex system that consistently shows aging-related decline across diverse individuals. Specifically, T-cell dynamics, capturing both thymic involution and lifelong antigenic exposure, provide insights into immune system aging. Although existing aging clocks, such as those based on DNA methylation (i.e., Horvath’s and GrimAge), offer valuable predictions of biological age and disease risk, these methods are often limited in ability and cost to reflect real-time immune function. We also explore cutting-edge techniques to measure T-cell states, such as flow cytometry, single-cell omics, cytometry by time-of-flight (CyTOF), and the potential of non-invasive retinal imaging, but these techniques also face these limitations. To account for the challenges with the above-mentioned methods, we propose the naive-to-exhausted T-cell ratio as a promising, quantifiable metric of immune aging. The conceptual framework benchmarks the naive-to-exhausted T-cell ratio against established epigenetic clocks, generating an “immune age curve” that offers clinicians and researchers a practical approach to integrate immune aging assessments into clinical and preventative care. To test our hypothesis, we conducted survival association analysis based on naive-to-exhausted T-cell levels across all major cancers (including adrenal carcinoma (HR = 0.19 (CI [0.082, 0.44]), *p* = 1.92e-5), low-grade glioma (HR = 0.47 (CI [0.33, 0.67]), *p* = 2.4e-5), and sarcoma (HR = 0.52 (CI [0.35, 0.77], *p* = 0.000984)). The survival analysis shows that a higher ratio of naive to exhausted T cells is associated with significantly better overall survival rates, with hazard ratios (HRs) ranging from 2.7e-9 to 0.7. These preliminary results support the predictive value of naive-to-exhausted T-cell levels for biological aging and disease progression prediction across multiple organ systems.

## 1 Introduction

The global demographic pyramid is inverting, with a growing proportion of older individuals compared to younger populations. In the United States, for example, the number of adults aged ≥ 65 years is expected to increase from 58 million to 82 million between 2022 and 2050—a 47% increase (Mather and Paola, 2024). Globally, during the same period, this specific population is expected to grow from 17% to 23% (Mather and Paola, 2024). This rise in the aging population is accompanied by physiological changes, including a decline in muscle mass, loss of strength, and a reduction in physical activity, increasing the risk of chronic diseases such as diabetes, cardio- and cerebrovascular disease, and cancer (Suryadinata et al., 2020). Neurological and psychological diseases such as Alzheimer’s, Parkinson’s, depression, and other psychiatric disorders also show an increased incidence in the aging population (Steverson, 2024). These trends impose a substantial burden on healthcare systems and national economies, necessitating immediate solutions to accurately forecast individuals’ risk for various aging-related diseases.

Over the past 2 decades, biological age estimation has emerged as a promising surrogate biomarker to assess biological aging and disease risk. Among the various biological systems, the immune system, particularly T-cell dynamics, has gained traction for its potential to measure immunological aging. While chronological aging is easily quantified based on numerical age, biological age reflects the physiological state, including immune system decline. However, quantifying immunosenescence and immune exhaustion remains a major gap in aging research, and more accurate measures of quantified biological age could enable earlier, targeted interventions to decelerate or reverse immune aging.

To address this, researchers have developed quantitative biomarkers based on physical, molecular, and cellular characteristics, commonly referred to as “aging clocks.” These clocks integrate clinical, genetic, and molecular data, such as telomere length, DNA methylation patterns, and transcriptomic, proteomic, and metabolite profiles (Hillary et al., 2020). These clocks can predict biological age with an error ranging from 3 (Lu et al., 2019) to 23 years (Karlsson et al., 2008), and can assess chronic disease risk. First-generation aging clocks primarily estimate the biological age, while subsequent generations, such as GrimAge, also predict disease onset and mortality risk by incorporating CpG methylation, smoking history, and circulating protein levels. GrimAge has demonstrated predictive value for chronic obstructive pulmonary disease, type 2 diabetes, and ischemic heart disease (Hillary et al., 2020).

The capacity of aging clocks to move beyond simple biological age estimation toward forecasting disease onset represents a major advance, offering new opportunities for preventative healthcare strategies. However, it is important to recognize their limitations. Issues such as variability across populations, reduced predictive accuracy in old age, challenges in clinical applicability, and the high cost or complexity of molecular profiling remain barriers to widespread adoption (Bell et al., 2019). Moreover, the biological mechanisms linking these biomarkers to aging processes are not yet fully understood, raising concerns about interpretability and long-term clinical implementation (Moqri et al., 2023).

Beyond these molecular approaches, the immune system itself offers a dynamic readout of biological age as immune cells constantly interact with both internal and external stressors and serve as a living record of cumulative wear and tear (Goronzy and Weyand, 2019). Specifically, T-cell subset distributions, proliferative capacity, and exhaustion markers mirror the balance between immune resilience and decline (Akbar and Henson, 2011). Immune profiling differs from organ-specific biomarkers related to aging in that it reflects systemic aging processes, thereby offering insights into the body’s ability to mount protective responses against diseases. The state of the immune system links cellular-level changes to aging processes, providing new facets to the existing aging clocks.

Recent research has focused on immune cell dynamics for more precise estimations of biological age and disease risk compared to the initial analysis of organ-based disease/aging marker identification. Circulating immune cells—including T lymphocytes—represent the state of all organs, reflecting cumulative exposure to stress, inflammation, and cellular damage (Dhodapkar, 2024). The concept of immunosenescence links age-related immune decline to increased disease susceptibility, including higher cancer risk in biologically older individuals (Berben et al., 2021). Oxidative stress and cellular damage due to environmental and lifestyle factors further accelerate immune aging, creating a permissive environment for oncogenesis.

Within the adaptive immune system, T lymphocytes play a pivotal role in defense against infection and chronic diseases. With age, the thymus—the site of T-cell production—involutes, reducing the output of new T cells (i.e., naive T cells). Meanwhile, the population of antigen-experienced and differentiated T cells expands, exhibiting signs of exhaustion and progressive functional decline (Salam et al., 2013). This shift in T-cell composition contributes to reduced immune responsiveness, frailty, and impaired vaccine responsiveness in older adults.

On the intervention or treatment side, growing evidence suggests that biological age is modifiable, leading to the development of interventional studies directed toward slowing or reversing the aging process. Trials on caloric restriction, plant-based diets, regular exercise, metformin, and vitamin D3 supplementation have shown the potential to slow or reverse biological aging (Johnson et al., 2022). For example, the CALERIE trial demonstrated deceleration of biological aging through caloric restriction, while the ongoing TAME trial is evaluating the impact of metformin on aging markers using methylation and proteomic clocks (Johnson et al., 2022). While the data demonstrate that biological age can be influenced by lifestyle and therapeutic interventions, further research is needed to confirm the long-term clinical efficacy of these approaches (Johnson et al., 2022).

To optimize the translational potential of these interventions, we delve into a comprehensive understanding of the cellular and molecular hallmarks of biological aging. This article discusses various approaches to measuring aging and synthesizes evidence on hallmarks of immune aging, with a focus on T-cell subset redistribution. We discuss emerging methodologies for biological age estimation, including flow cytometry, single-cell omics, mass cytometry (cytometry by time-of-flight, CyTOF), and retinal imaging. By integrating these approaches, we aim to provide researchers and clinicians with a blueprint to quantify and ultimately slow human biological aging.

## 2 Currently established measures of aging

The emerging research field of gerontology focuses on identifying and understanding the aspects and drivers of the aging process based on the physical, mental, and cultural factors (Harris, 1988). Over the years, numerous approaches based on these factors have been developed to quantify aging beyond chronological age, aiming to track the physiological, molecular, and cognitive variables that evolve as individuals age, with varying degrees of precision and success. These approaches vary in accuracy and clinical utility, and this section summarizes them by reviewing the main classes of aging biomarkers and evaluating their contributions and limitations.

### 2.1 Physical aging markers

**FIGURE 1:**
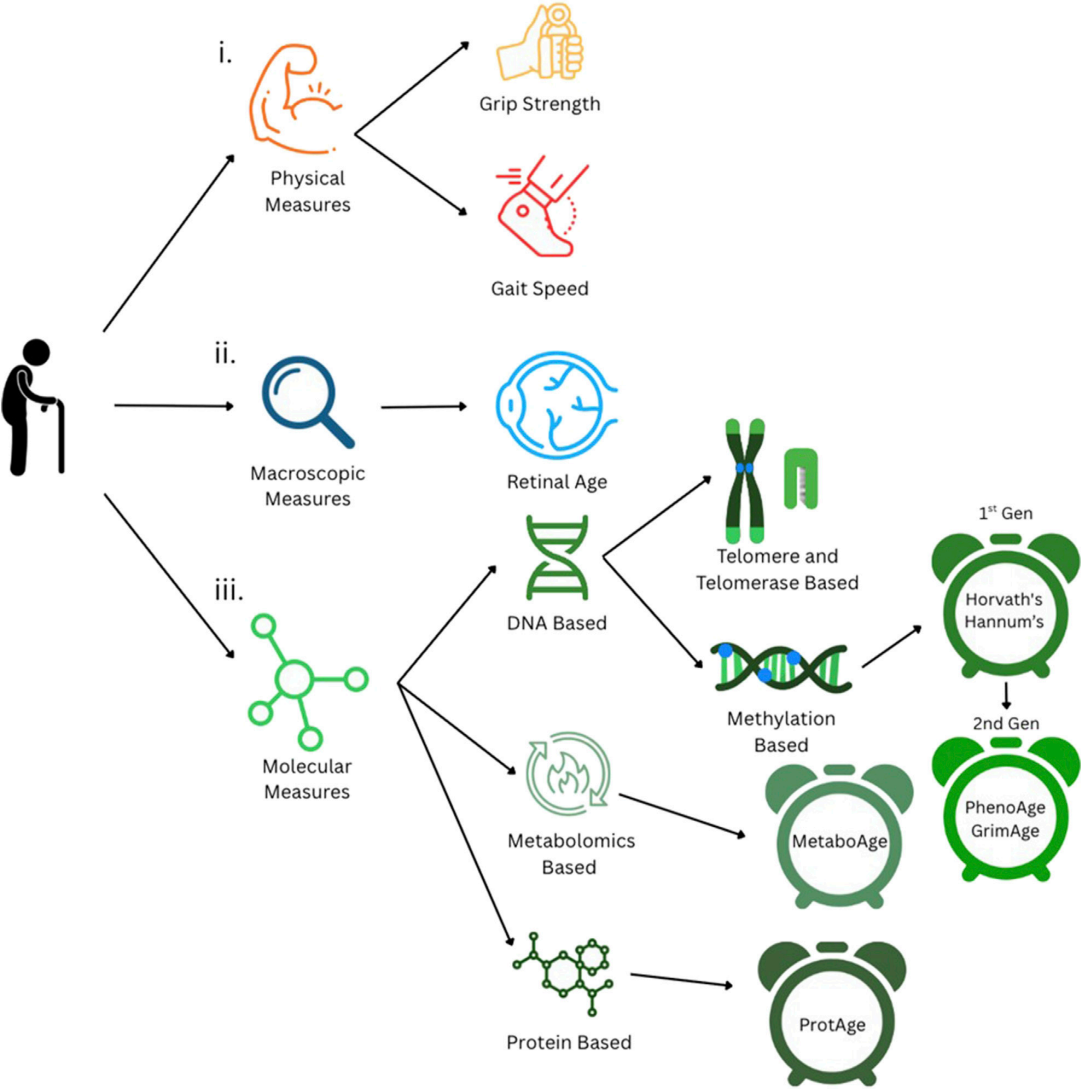
Currently established measures of biological aging. i. Physical measures of aging: grip strength and gait speed. ii. Macroscopic measures, such as retinal imaging-based age estimation. iii Molecular measures of aging, including DNA-, metabolite-, and protein-based methods.

Some of the earliest factors examined as biomarkers of aging centered on physical measures, such as gait speed and grip strength (Figure 1). These assessments are considered valuable, noninvasive indicators of muscle strength and functional decline. For example, decreased grip strength is associated with cognitive decline, diabetes, and early mortality (Peterson et al., 2023). Similarly, a lower gait speed is associated with a higher risk of mortality from certain conditions, such as chronic kidney disease (Zhang et al., 2025) and cardiovascular disease (Busch et al., 2015). Furthermore, links between declines in grip strength and gait speed have been observed to accelerate aging (Peterson et al., 2023; Rasmussen et al., 2019). While there are clear trends and associations between grip strength and chronic disease risk, grip strength has not yet been validated as a direct predictor of biological age (Kemala et al., 2025; Martin et al., 2015). Gait speed has been explored as a predictor of biological age, but its accuracy remains limited, with an average error margin of ±10 years (Kuroda et al., 2025).

Beyond these measures, clinical gerontology often incorporates activities of daily living (ADLs) and instrumental activities of daily living (IADLs) to assess an individual’s functional independence. These assessments quantify how age-related physical decline translates into limitations in everyday activities, such as personal hygiene and managing finances, and they are widely utilized in aging studies and clinical settings (Edemekong et al., 2025). Unlike isolated physical metrics, ADLs and IADLs provide a more holistic assessment of functional aging, especially when correlated with molecular parameters of aging. Recent studies show that markers of muscle function and activity can align with epigenetic clocks, which track biological age using DNA methylation signatures (Jokai et al., 2023). Emerging tools such as DNAmFitAge integrate physical fitness parameters (e.g., VO_2_ max, gait speed, and grip strength) into DNA methylation models, providing more accurate biological age predictions that link lifestyle factors to molecular decline.

### 2.2 Telomere-based aging measures

Molecular measures have also been considered biomarkers of aging. One of the earliest is telomere length, which is an indicator of chromosomal integrity (Figure 1). Telomeres are protective DNA-protein structures at the ends of chromosomes that shorten due to DNA replication (McHugh and Gil, 2018; Sfeir, 2012). This shortening, called telomere attrition, can be linked to the number of times a cell has divided, providing a proxy for biological age (Lundblad, 2012). However, telomere attrition is inconsistent across similarly aged individuals and tissues from the same individual (Pepke, 2024). This makes telomere attrition a relatively coarse measure of biological age, with notably large error margins, which can range from approximately ±10 to 23 years (Alhusseini et al., 2016; Karlsson et al., 2008).

Related to the telomere, another molecular biomarker of aging is telomerase activity (Figure 1). Telomerase is an enzyme that performs maintenance on telomeres (He and Feigon, 2022), and its activity declines with age in the majority of cells (Iwama et al., 1998), making it a potential marker of biological aging. However, its predictive accuracy is limited due to large variations in the telomerase activity across cell types and states (Lin et al., 2010).

### 2.3 DNA methylation clocks

Molecular markers of biological aging then advanced to DNA methylation, which has emerged as one of the most promising methods for estimating biological age (Figure 1). DNA methylation, as a process, involves the addition of a methyl group (CH_3_) to specific cytosine nucleotides near promoter regions in the DNA sequence (called CpG sites) (Moore et al., 2013). This methylation prevents transcription factors from binding to transcription start sites and transcribing genes (Moore et al., 2013), resulting in suppressed gene expression. Methylation analysis has shown that different genomic sites exhibit alterations in methylation patterns with age, regulating various biological processes and pathways (Salameh et al., 2020). DNA methylation, over time, can be measured through methylation arrays or bisulfite sequencing that track gene-specific methylation and correlate these profiles with age.

DNA methylation was utilized in the so-called Horvath’s clock to predict biological age based on the methylation patterns of 353 CpG sites, 193 of which gain methylation and 160 of which lose methylation with age (Horvath, 2013). Horvath’s clock can predict the age of multiple tissues and has shown that it can measure the accelerated aging that occurs in diseases such as cancer (Horvath, 2013), with a margin of approximately 3 years. Similarly, Hannum’s clock was also developed based on the DNA methylation of 71 CpG sites in whole blood and accounts for individual gender and genetic variability (Hannum et al., 2013). However, these first-generation clocks, such as Horvath’s and Hannum’s clocks, have limited utility for chronic disease prediction and do not provide assessments that correlate with clinical outcomes, such as mortality and disease progression.

To further enhance the significance of these aging clocks, second-generation clocks have been developed that provide information on biological aging and the risk of onset and progression of chronic diseases. PhenoAge and GrimAge are two highly popular second-generation biological aging clocks. PhenoAge estimates biological age and disease risk by analyzing the DNA methylation patterns of 513 CpG sites in blood samples, which are associated with biological age and the onset of chronic diseases, such as cancer and Alzheimer’s disease (Levine et al., 2018). It can also provide insights into immunosenescence, which refers to the age-related decline in immune system effectiveness driven by chronic inflammation and thymic involution (Liu et al., 2023).

On the other hand, the GrimAge epigenetic clock was specifically developed to detail mortality and morbidity by measuring 1,030 CpG sites that are surrogates for various plasma proteins related to aging (Lu et al., 2019). This clock can predict biological age with an error margin of 3 to 5 years, allowing the estimation of the onset of certain diseases and events, such as death, coronary heart disease, and cancer (Lu et al., 2019). To further improve its accuracy, GrimAge 2.0 was developed by including two additional plasma proteins, allowing for accurate age prediction in younger individuals and robust performance across diverse ethnic groups (Lu et al., 2022).

### 2.4 Metabolomic and proteomic aging measures

While DNA methylation-based clocks provide valuable insights into biological age, chronic diseases, and clinical outcomes, they fall short in capturing the direct mechanistic and functional drivers of aging biology. To achieve more precise and informative predictions, it is necessary to move beyond the epigenomic layer and develop predictors rooted in the functional and mechanistic processes, which are often directly related to the physical and biological changes observed in aging. Keeping this in mind, researchers have explored the proteomic (Argentieri et al., 2024) and metabolomic (Van Den Akker et al., 2020) (Figure 1) layers of the genome to predict biological age and disease-specific risk with greater accuracy. As a step in this direction, Van Den Akker et al. (2020) developed MetaboAge, a biological aging model based on the levels of 56 metabolites measured in 18,716 samples. The MetaboAge model calculates the difference between the predicted biological age and chronological age (i.e., ΔMetaboAge) (Van Den Akker et al., 2020), with a higher difference indicating a higher biological age and poorer cardiometabolic health (Van Den Akker et al., 2020). Conversely, a lower ΔMetaboAge value indicates a low biological age associated with better cardiometabolic health (Van Den Akker et al., 2020).

Similarly, investigators developed ProtAge, a model that estimates biological age based on the expression of 204 proteins (Argentieri et al., 2024). This model predicts biological age with an average error of ±5.7 years and calculates the ProtAge gap, which is defined as the difference between an individual’s proteomic and chronological age (Argentieri et al., 2024). Beyond estimating the biological age, ProtAge also provides insights into chronic disease risk. Increases in proteomic age, or a larger ProtAge gap, are associated with a higher incidence of cardiovascular, hepatic, renal, and pulmonary diseases, diabetes, neurodegenerative conditions, and cancer (Argentieri et al., 2024).

### 2.5 Imaging-based non-invasive biomarkers

All of the former modern molecular predictors provide valuable data regarding biological age and age-related chronic disease risk and onset, but they require invasive tests. Thus, non-invasive measures such as retinal imaging have emerged as accessible tools for assessing an individual’s biological age without invasive sampling (Figure 1). Retinal imaging achieves this by capturing subtle age-related changes in the microvascular and neural structures of the eye that can then be related to biological age. The viability of retinal age as a biomarker stems from the retina’s shared origin with the central nervous system and from its microvascular structure, which is closely related to that of the kidneys and the brain (Grimbly et al., 2024). This structure can reflect systemic aging processes, including vascular stiffness, inflammation, and neurodegeneration. Recent advances in deep learning have enabled the development of algorithms trained on fundus photographs, which can predict biological age with high degrees of accuracy. The discrepancy between the predicted retinal age and chronological age, which is referred to as the “retinal age gap,” has been associated with an increased risk of cognitive decline, cardiovascular disease, and all-cause mortality (Zhu et al., 2023). As a quick and low-cost approach, retinal imaging holds promise for integrating biological age screening into routine clinical checkups, particularly in populations where genomic assays are less accessible (Yu et al., 2025).

### 2.6 Organ-specific aging trajectories

Although the above-described approaches have been able to predict biological age with varying degrees of accuracy, they have all relied on singular factors and organ systems when developing prediction models. However, this may not be the best approach, as it assumes that all organs age linearly at the same rate. Thus, we propose enhancing approaches focused on a single biological element by integrating health metrics across multiple organ systems, which may provide a more accurate assessment of biological age. This concept has been demonstrated in a mouse model, where RNA and proteomics analyses of 17 organs at 10 distinct life-cycle stages revealed that aging induces organ-specific alterations in both gene expression and protein abundance (Schaum et al., 2020). It can, therefore, be gleaned that protein and gene sets related to aging behave differently (with accelerated/decelerated rates of change) across different organs, indicating that a one-size-fits-all test may not be optimal (Schaum et al., 2020), at least in mice. These organ-specific trajectories suggest that current human clocks based on a single factor may not be as accurate as an organ-by-organ approach.

## 3 Novel cellular- and immune-based measures of aging

### 3.1 Introduction to immune aging

In recent years, the immune system has been studied as a promising avenue for assessing aging because the immune system is influenced by alterations in diverse cell types across all organ systems. Functioning as the body’s primary defense mechanism, the immune system undergoes rapid changes across multiple body systems. Unlike static molecular markers, immune aging can capture these dynamic, cumulative changes, which are shaped by both intrinsic decline and external exposures, thus offering a broader window into whole-body aging.

Age-related shifts occur across the multicomponent immune system, which comprises the various immune cell types, including monocytes, macrophages, B cells, and T cells. Monocytes and macrophages play a critical role in modulating the immune clearance of pathogens and cancer cells, undergo transcriptional reprogramming via epigenetic modifications, and show a decrease in phagocytic capacity with age (De Maeyer and Chambers, 2021). Similarly, B cells, which are responsible for antibody production, appear in lower concentrations and exhibit decreased antibody diversity with age (De Mol et al., 2021). However, T cells are key to adaptive immunity, and develop and mount cytotoxic responses against pathogenic and self-altered antigens to eliminate them, while also generating long-lasting memory for sustained immune defense. Additionally, the balance between T-cell populations (i.e., naive, effector, and exhausted) in the immune system stands out as one of the most robust and quantifiable measures of system-level immune aging. However, it is important to note that T-cell differentiation states follow a spectrum and cannot be merely categorized as naive, effector, or exhausted subtypes. Effector cells are all antigen-experienced: some are activated, some are in resting memory states, and some are exhausted or immunosenescent. The spectrum of states and their implications for aging are important considerations for future research.

### 3.2 T-cell dynamics as biomarkers of aging

T cells are central to adaptive immunity and immune memory, and their dynamics are increasingly being studied as biomarkers of biological aging. They exist in three main functional states, each of which aligns with a different stage of their lifespan: naive, effector, and exhausted (Tough and Sprent, 1995). Naive T cells are generated in the thymus and represent a blank slate that is capable of responding to novel antigens. Upon encountering a pathogen, naive T cells differentiate into effector T cells, which mediate immune responses such as pathogen and infected cell clearance. After completing their cytotoxic function, some effector T cells transition to an exhausted state, which is characterized by functional decline and reduced proliferative capacity.

The exhausted T-cell phenotype typically arises in settings of chronic antigenic stimulation, such as persistent viral infections or the tumor microenvironment, where sustained TCR signaling drives the upregulation of inhibitory receptors, including PD-1, TIM-3, and LAG-3 (Baessler and Vignali, 2024). This chronic activation promotes epigenetic and transcriptional reprogramming that diminishes cytokine secretion, proliferation, and cytotoxicity (https://pmc.ncbi.nlm.nih.gov/articles/PMC8005453/?utm). Exhausted T cells contribute to disease progression by impairing immune surveillance and causing inflammation that promotes the aggressiveness of chronic diseases (Chow et al., 2022).

**FIGURE 2:**
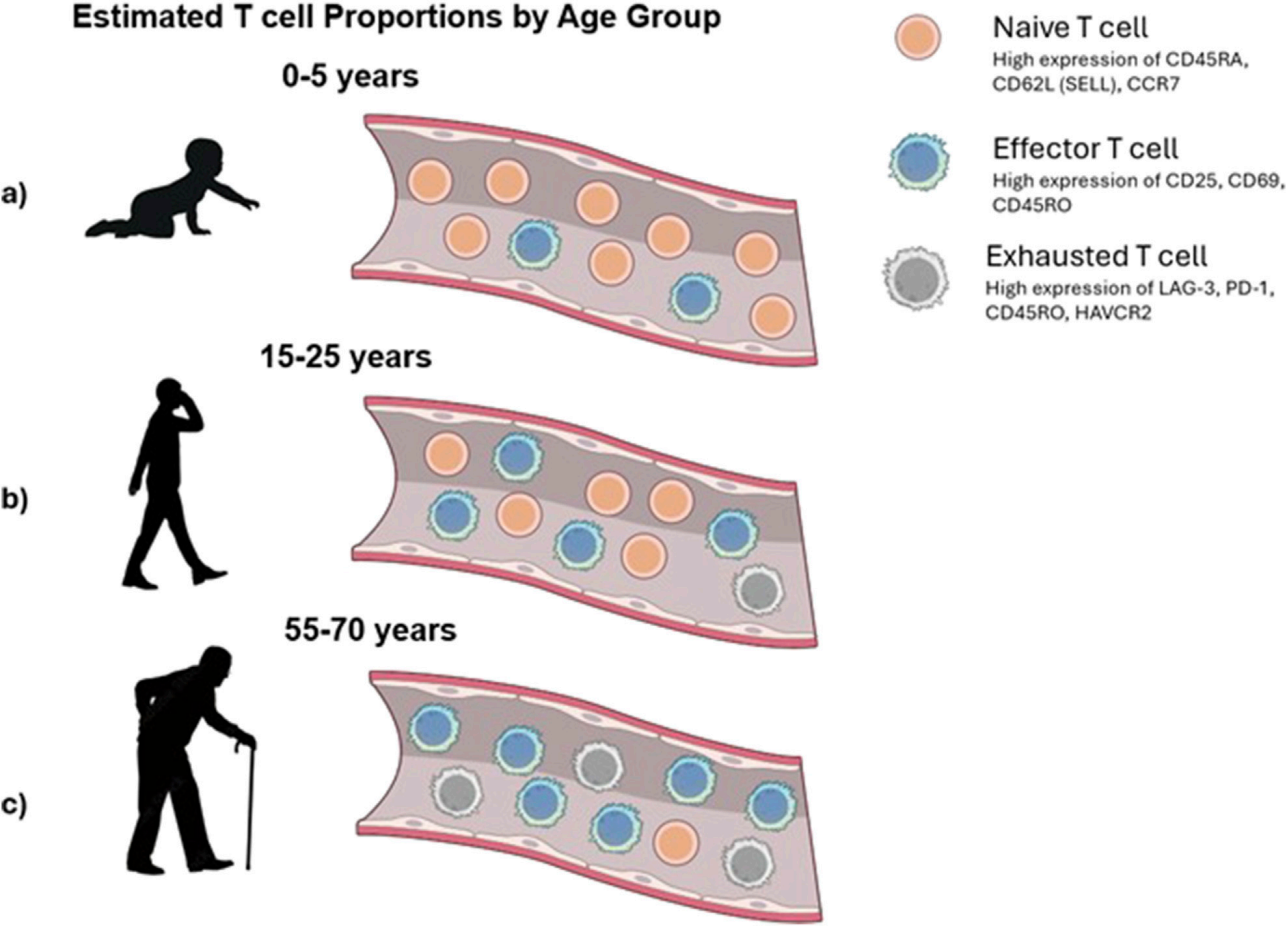
Conceptual view of changes in T-cell populations with age. **(a)** Infants exhibit an abundance of naive T cells that have not yet encountered antigens. **(b)** In adults, the T-cell compartment typically maintains a balanced composition of naive and antigen-committed effector T cells, enabling robust immune responses. **(c)** Elderly individuals experience a marked decline in naive T cells and an accumulation of exhausted and late effector T cells.

The proportions of T cells in these distinct stages shift with age. For example, infants and young children possess a large pool of naive T cells, but individuals over 80 years of age exhibit a smaller proportion of naive T cells and higher concentrations of terminally exhausted T cells (Li et al., 2019) than younger individuals (Figure 2).

**FIGURE 3:**
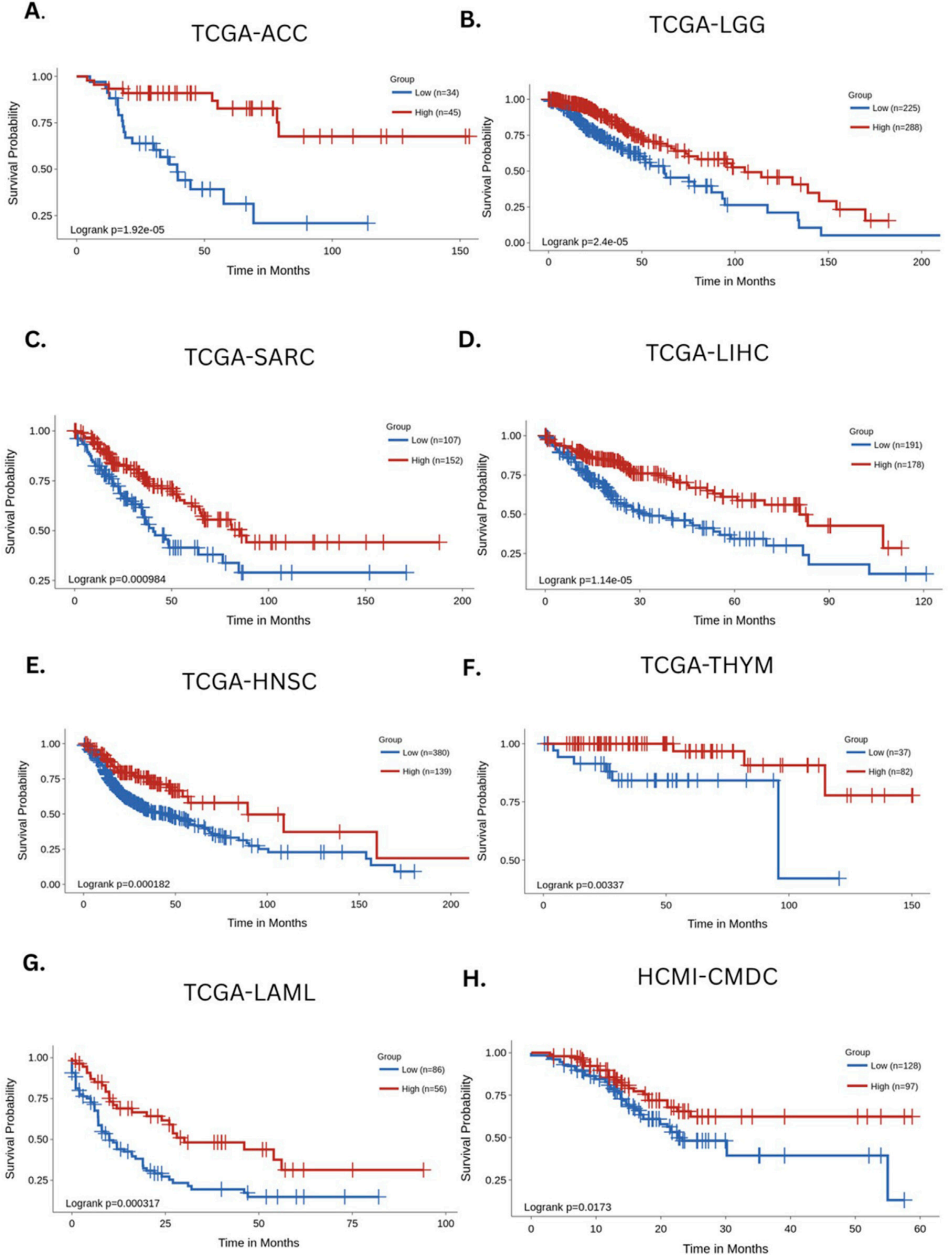
Survival analysis based on the estimated levels of naive and exhausted T cells across major cancers. The Kaplan–Meier curves depict the survival association of naive/exhausted T-cell levels across the following cancers: **(A)** TCGA-ACC (adrenocortical carcinoma), **(B)** TCGA-LGG (low-grade glioma), **(C)** TCGA-SARC (sarcoma), **(D)** TCGA-LIHC (liver hepatocellular carcinoma), **(E)** TCGA-HNSC (head and neck cancer), **(F)** TCGA THYM (thymic tumors), **(G)** TCGA-LAML (acute myeloid leukemia), and **(H)** HCMI-CMDC (pan-cancer samples) (Unal, 2017).

As an example of the clinical implications of naive and exhausted T-cell distributions, we performed a novel survival analysis using publicly available cancer datasets from The Cancer Genome Atlas (TCGA) and the Human Cancer Models Initiative (HCMI) Cancer Model Development Center (CMDC). Using the gene contrast module within the Survival Genie platform (Dwivedi et al., 2022), we compared the survival outcomes of patient samples that were stratified based on the relative expression of gene markers associated with naive T cells (*PTPRC [CD45]*, *CCR7*, and *SELL[CD62L]*) and exhausted T cells (*HAVCR2 [TIM-3]*, *CD279 [PDCD1]*, and *LAG3).* The cumulative enrichment scores for naive and exhausted T-cell signatures were computed using the gene set variation analysis (GSVA) algorithm (Hänzelmann et al., 2013). Based on these GSVA-derived scores, the samples were stratified into high and low groups using an optimal cut-point selection approach (Unal, 2017). The significance of the association was calculated using the log-rank *p*-value. Additionally, for each dataset, we evaluated survival associations by applying a Cox proportional hazards model (Abd ElHafeez et al., 2021), which estimates hazard ratios (HRs) while accounting for time-to-event and censored survival data. In this analysis, an HR < 1 indicates that a higher naive-to-exhausted T-cell ratio is associated with improved patient survival, whereas an HR > 1 suggests that a higher ratio is associated with worse survival outcomes (Figure 3).

Across multiple cancer types, a higher ratio of naive-to-exhausted T cells exhibited an association with significantly better survival, including adrenal carcinoma (HR = 0.19 (CI [0.082, 0.44]), *p* = 1.92e-5, Figure 3A), low-grade glioma (HR = 0.47 (CI [0.33, 0.67]), *p* = 2.4e-5, Figure 3B), sarcoma (HR = 0.52 (CI [0.35, 0.77], *p* = 0.000984, Figure 3C), liver hepatocellular carcinoma (HR = 0.46 (CI [0.32, 0.65]), *p* = 1.14e-5, Figure 3D), head and neck cancer (HR = 0.52 (CI [0.37, 0.74], 0.000182, Figure 3E), thymic tumors (HR = 0.15 (CI [0.038,0.64]), *p* = 0.00337, Figure 3F), acute myeloid leukemia (HR = 0.44 (CI [0.28, 0.7]), *p* = 0.000397, Figure 3G), and multiple cancers (HR = 0.56 (CI [0.35, 0.91]), *p* = 0.0173, Figure 3H). The Kaplan–Meier survival curves consistently demonstrated that patients with a higher ratio of naive-to-exhausted T-cell markers had significantly improved overall survival. On the other hand, a lower naive-to-exhausted T-cell ratio, indicating higher levels of exhausted T cells, is associated with poorer overall survival. This supports the hypothesis that the naive-to-exhausted T-cell ratio is a prognostic factor for survival across cancers and might represent cancer-associated accelerated aging. Therefore, we hypothesize that the above-mentioned ratio could be a promising biomarker of biological aging and the immune system’s competence in the context of cancer. Individuals with higher proportions of exhausted T cells may be at greater risk of chronic disease incidence and progression, whereas those with higher proportions of naive T cells can generate diverse T-cell pools directed against aging processes and disease-causing cellular alterations (see Supplementary Figures S1 and S2 for additional analyses).

Furthermore, survival analysis was performed on the diverse pan-cancer dataset HCMI-CMDC, which contains data from tumors, neoplasms, gliomas, carcinomas, and other cancers in 32 different body areas. This provided a benchmark for survival outcomes based on the naive-to-exhausted T-cell ratio in a single dataset encompassing multiple cancers from diverse tissues of the human body. The results of the survival analysis indicated a strong association between the naive-to-exhausted T-cell ratio and patient survival (Figure 3H).

As patients age, thymic involution leads to diminished naive T-cell output, while chronic antigenic exposure contributes to the accumulation of exhausted T cells. These exhausted cells, through impaired effector functions and sustained inhibitory signaling, weaken tumor and pathogen control while also driving chronic inflammation. A low naive-to-exhausted T-cell ratio, therefore, reflects systemic immune decline, diminishing the body’s capacity to control malignancies and increasing vulnerability to cancer progression. These findings strongly support recent technological advancements that have made it possible to measure T-cell proportions more accurately and characterize T-cell dynamics at the single-cell level.

Although the study evaluates T-cell proportions as an indicator of immune aging, immunosenescence extends to other immune compartments. With increasing age, B cells exhibit reduced diversity and impaired antibody responses (Li et al., 2023), NK cells demonstrate altered cytotoxicity (Fu et al., 2025), and monocytes/macrophages show “inflammaging” (Wrona et al., 2024). Together, these changes contribute to immune aging and may be integrated into composite immune clocks. Future frameworks could combine T-cell ratios with these other immune signatures mentioned above to generate more comprehensive predictors of biological age and disease risk.

### 3.3 Techniques for measuring T-cell states

In order to understand the field of immune aging, technologies must be able to capture the diversity of T-cell states, specifically naive, effector, and exhausted T-cell phenotypes. These techniques work to offer avenues into clinical translation, each with advantages in resolution, throughput, and feasibility. This section details techniques to identify and quantify T-cell states, which, in combination, could form a framework for creating an immune age curve to determine biological age.

#### 3.3.1 Flow cytometry and fluorescence-activated cell sorting

Flow cytometry—specifically, fluorescence-activated cell sorting (FACS), which uses fluorescently conjugated antibodies to quantify surface protein expression across cell populations—has long served as the gold standard for identifying immune cell subtypes based on surface or intracellular protein expression (Flow Cytometry, 2014) (Supplementary Figure S3). This technique is the most common method for immune cell phenotyping that is clinically adopted. By labeling cells with fluorescently conjugated antibodies and passing them through a flow cytometer, researchers can sort and quantify populations of naive, effector, and exhausted T cells (Lugli et al., 2017). Modern high-parameter flow cytometers equipped with multiple lasers enable simultaneous detection of 12+ markers, allowing for complex phenotyping in a single assay (Kwok et al., 2023). For example, Lugli et al. (2017) used multicolor FACS panels to identify T-cell subsets based on CD45RA, CCR7, CD27, and PD-1 expression. However, despite its sensitivity and speed, flow cytometry has limited multiplexing capabilities and requires substantial operator expertise to correctly quantify T-cell subsets (Flow Cytometry, 2014).

#### 3.3.2 Single-cell omics

To capture transcriptional signatures beyond targeted marker expression, single-cell RNA sequencing (scRNA-seq) has emerged as a promising approach (Supplementary Figure S4). scRNA-seq allows measurement of the expression of 2,000–3,000 genes in individual cells to identify T cells and their subsets and assess associations with accelerated aging (Abondio et al., 2022). Over the past two decades, single-cell profiling has been extensively implemented to understand the mechanisms of accelerated aging, but the technology is still far from clinical implementation due to high cost, data sparsity, and complex analytical requirements. Multi-omics single-cell approaches can simultaneously measure transcriptomic, epigenomic, and proteomic profiles, providing a holistic picture of how T-cell phenotypes evolve with age (Lee et al., 2020).

#### 3.3.3 Cytometry by time-of-flight

CyTOF bridges the gap between surface phenotyping and high-dimensional data integration by overcoming the limitations of flow cytometry and offering improved multiplexing capabilities (Supplementary Figure S5). Using metal isotope-tagged antibodies, CyTOF can simultaneously quantify over 40 markers per cell (Tirosh et al., 2016), allowing for an in-depth characterization of T-cell states, including exhaustion and activation. Lin et al. (2018) demonstrated the utility of CyTOF using a novel magnetic enrichment technique coupled with traditional CyTOF to profile T-cell populations. Nonetheless, CyTOF’s high instrumentation cost and complex data analysis requirements currently limit its clinical implementation.

### 3.4 Retinal imaging as a non-invasive proxy for immune aging

While the technologies described above rely on blood-based sampling, retinal imaging offers a promising, noninvasive alternative to assess immune aging. The eye, particularly the retinal vasculature, is sensitive to age-related microvascular and immune changes, so retinal research can be applied to age-related damage observed in other comorbidities such as diabetes, cardiovascular disease, and neurodegeneration. Clinically, ophthalmic imaging is already used to assess vascular integrity through tools such as adaptive optics scanning laser ophthalmoscopy (AOSLO). AOSLO enables direct visualization of single immune cells moving through retinal capillaries *in vivo*, providing readouts of cell density, motility, and vessel wall interactions (Joseph et al., 2020). These parameters are particularly relevant to immunosenescence because, in healthy young individuals, naive T cells and other circulating leukocytes typically show high motility and transient vascular interactions, reflecting a dynamic surveillance state (Torres et al., 2023). With increasing age, however, thymic involution and cumulative antigen exposure reduce the naive pool and expand the proportion of exhausted immune cells. These cells often display reduced motility, altered adhesion to vascular endothelium, and impaired clearance from tissues (Xu et al., 2024). AOSLO can capture these hallmarks of immune aging in real time by quantifying changes in immune cell transit speed and clustering patterns within the retinal microvasculature (Joseph et al., 2020). Given its shared embryologic origin and structural similarities with cerebral vessels, the retinal vasculature could provide insights into systemic microvascular and immune health (Tao et al., 2022). This suggests that longitudinal AOSLO imaging could serve as a surrogate marker for broader age-related changes throughout the body. Since these datasets can be acquired repeatedly without invasive sampling, they offer a clinically feasible way to monitor immunosenescence *in vivo*, particularly in clinical settings, where frequent blood draws may be impractical.

When paired with AI-driven analysis, such as deep learning models trained on fundus photographs (Zhu et al., 2023) to predict all-cause mortality, retinal imaging could contribute to a composite aging clock that integrates immune, vascular, and neurodegenerative signals. By training algorithms to recognize changes in immune cell dynamics that correlate with T-cell exhaustion or decreased naive output, AOSLO could bridge the gap between molecular immunology and non-invasive screening. However, retinal scanning-based aging detection remains a developing field, requiring further validation before it can reliably serve as a standalone proxy for immune age in clinical practice. Together, the technologies listed above represent a spectrum of approaches to mapping the immune architecture of aging, and taking advantage of these methods could contribute to an immune age curve that accurately estimates biological age.

All the techniques mentioned above, including flow cytometry, single-cell RNA sequencing, and CyTOF, classify immune cell states based on the expression of selected markers. Although these marker-based approaches provide a useful, albeit indirect, measure of cell states, they are subject to certain limitations and errors. Definitive confirmation of T-cell exhaustion requires functional assays involving antigen stimulation followed by the measurement of cytokine or chemokine secretion. However, implementing these assays on a large scale is not feasible, and minimal data are available to support the development of a biological aging clock using these approaches. Therefore, in this preliminary study, we focused on developing a predictive biological clock by correlating the immune state, measured based on gene/protein expression, with biological age, rather than delving deeper into functional/metabolic T-cell exhaustion.

## 4 Immune-derived estimation of biological age

### 4.1 Rationale for immune-based aging metrics

Traditional biological age estimation methods, such as DNA methylation-based clocks (i.e., Horvath’s clock or GrimAge), have contributed significant results for future aging research. However, these current approaches are often hindered by limitations such as cost, complexity, tissue specificity, and machinery, which limit their broader applicability. Furthermore, many of these clocks capture static snapshots, which may fail to reflect real-time changes in the immune composition.

The immune system emerges as an alternative to capture the interactions between intrinsic aging processes and environmental exposures. The composition of the immune system undergoes relatively quantifiable transformations with age across individuals through immunosenescence, such as thymic involution, chronic antigenic stimulation, and the shift from naive to exhausted T-cell phenotypes due to low-level chronic inflammation. As this ratio changes with immune system variability—referred to as immunosenescence—the ratio of naive to exhausted T cells holds promise as a universal biomarker of biological aging.

### 4.2 T-cell ratio as an aging indicator

As discussed earlier, the naive-to-exhausted T-cell ratio is prognostic for survival in the majority of the cancers tested in the TCGA and HCMI-CDMC datasets. We posit the naive-to-exhausted T-cell ratio as a quantitative metric for immune aging.

Naive T cells can express CD4 or CD8, distinguishing them as helper or cytotoxic T cells, but they are further defined by the presence or absence of specific markers. In particular, CD27, CD45RA, CCR7, and CD62L serve as markers that separate naive cells from effector and exhausted subtypes. Intracellular transcription factors such as FOXP1, ZEB2, and TBX21 (Tao et al., 2022) can also mark naive and effector T-cell differentiation states. For example, during differentiation, T cells lose CD27 expression, thus identifying cells with a high expression of CD27 as a positive marker of naive T cells. Similarly, combinations such as CD45RA^+^, CCR7^+^, and CD62L^+^ mark naive T cells (Tirosh et al., 2016). With age, thymic involution reduces the output of these naive populations, and T cells subjected to chronic stimulation can enter a state of exhaustion characterized by sustained expression of inhibitory receptors, including PD-L1^+^, TIM3^+^, and LAG3^+^, which are connected to impaired immune responses and chronic inflammation (Qi et al., 2025). This phenotype is typically triggered by persistent immune activation and the cumulative burden of lifetime immune challenges (immunobiography). The expansion of exhausted T cells is linked to increased infection risk, poor chronic disease outcomes, including cancer, diabetes, and vascular diseases, and frailty in older adults. This age-related shift in T-cell dynamics represents a bottleneck in immune responses to pathogens, linking exhaustion to biological aging. By calculating the ratio of these two populations, we obtain a single metric that reflects both the decline in thymic regenerative potential and the increase in immune dysfunction. Importantly, this metric is not limited to oncology; its trajectory may differ in infectious diseases and autoimmune disorders, highlighting its potential as a generalizable biomarker of immune aging. Since this ratio accounts for the two mechanistic hallmarks of biological aging (the loss of naive T-cell output and the gain of exhausted T cells), it may outperform other biomarkers in predicting immune decline and mortality risk.

### 4.3 Conceptual framework for the immune age curve

To construct an immune age curve, we will collect and integrate publicly available gene and protein expression datasets spanning a wide range of ages, including infants, toddlers, adults, and elderly individuals. Within each dataset, we will identify and quantify markers associated with naïve and exhausted T-cell populations. Using these markers, we will compute the average expression values or module scores (Tirosh et al., 2016) to estimate the relative abundance of each cell state. These quantitative measures will serve as the foundation for developing an immune aging curve.

As an initial approach, we will fit a regression curve between the estimated ratio of naïve to exhausted T cells and chronological age. This baseline model will provide a simple predictor of biological age, enabling estimation when the relative levels of these T-cell subsets are known (i.e., naïve and exhausted T cells). However, to achieve greater accuracy and capture nonlinear patterns, we will also develop machine learning-based predictors (Qi et al., 2025). These models will be trained using the same immune features, with the datasets divided into training and validation cohorts to ensure generalizability. Model performance will be rigorously evaluated using metrics such as the mean absolute error (MAE) and correlation with chronological age (Schneider and Xhafa, 2022).

If the machine learning model outperforms the linear regression predictor, this will be adopted as the preferred framework. Ultimately, this approach will allow us to build an accurate immune age clock that not only estimates biological age more precisely but also provides insights into immune system dynamics across the human lifespan.

### 4.4 Benchmarking against methylation-based clocks

To validate the immune-based biological age model, it should be benchmarked against established DNA methylation clocks, particularly GrimAge, which is a second-generation epigenetic clock trained to predict morbidity and mortality risk by incorporating CpG methylation sites (Moore et al., 2013). Since GrimAge is strongly correlated with systemic inflammation, immunosenescence, and time to disease onset, it serves as an ideal comparator for immune-derived metrics of biological age. Correlating immune biological age with GrimAge and measuring the gap between the two would allow researchers to assess whether T cell-based markers reflect or diverge from epigenetic markers of systemic aging.

## 5 Conclusion

As global life expectancy increases, the proportion of individuals over the age of 65 is growing rapidly. This demographic shift has brought age-related diseases to the forefront of public health concerns, burdening healthcare systems and economies. In this context, there is a growing need for accurate, individualized biomarkers of biological aging that can predict disease onset, monitor treatment outcomes, and identify health risks beyond chronological age.

Existing aging clocks, such as those based on methylation and metabolic profiles, offer deeper insights into individual aging, but they often fail to reflect dynamic physiological changes. Many are tissue-specific, costly, or lag behind real-time immune shifts. However, the immune system, particularly the composition of T-cell populations, stands out as an effective marker of biological aging. The immune system provides a unique perspective on aging across organ systems due to the circulation of immune cells throughout the body. Over time, predictable changes in T-cell proportions lead to a decline in naive T cells and the accumulation of exhausted T cells. These cells are sensitive to internal and external exposures, which can accelerate an individual’s biological age.

Multiple high-resolution technologies, such as flow cytometry, mass cytometry (CyTOF), and single-cell multi-omics, already enable the quantification of T-cell phenotypes at the single-cell level. These platforms allow researchers to profile surface markers associated with naive (CD45RA^+^ and CCR7^+^), effector, and exhausted (PD-1^+^, TIM-3^+^, and LAG-3^+^) T cells. At the same time, the development of non-invasive imaging technologies, such as AOSLO, coupled with AI-driven computational modeling, offers a translational path for bringing immune aging metrics into clinical practice. These tools could enable outpatient-friendly, real-time monitoring of immune age without invasive sampling, thus broadening access to preventative screening.

To validate the clinical relevance of T cells as predictors of mortality, we conducted a survival analysis using publicly available datasets from TCGA and HCMI-CMDC. Through the Survival Genie platform, we stratified patients by gene expression signatures associated with naive and exhausted T cells, confirming that higher expression of naive T-cell markers correlated with improved survival across multiple cancers. In contrast, increased exhausted T-cell markers were associated with poorer outcomes. These findings support the potential of the naive-to-exhausted T-cell ratio as a predictor of immune aging and disease vulnerability.

As a result of this analysis, we recommend future studies to refine and standardize immune aging clocks based on T-cell ratios. This indicator shows promise due to its ability to integrate multiple important features of immunosenescence, including thymic involution and lifetime antigenic load. Future research should focus on the development of machine learning models to generate immune age curves, benchmarked against validated epigenetic clocks such as GrimAge. Other established parameters, such as memory subsets and senescent T cells, will be taken into consideration in future studies after the development of the immune age curve. If this comparative analysis enhances our study, we will combine these parameters into the final algorithm.

When combined with non-invasive imaging and AI-based predictive models, these immune clocks could provide clinicians with powerful tools for early disease detection, individualized risk stratification, and preventative interventions. Ultimately, combining immune-based and non-invasive tools may revolutionize gerontology and preventative medicine.

## Data Availability

The original contributions presented in the study are included in the article/Supplementary Material, further inquiries can be directed to the corresponding authors.

## Supplementary material

SUPPLEMENTARY FIGURE S1 Survival analysis by deconvoluting the proportions of naive and exhausted T cells across major cancers. The analysis was performed by calculating the levels of naive T cells based on a positive gene signature (PTPRC, *CCR7*, and *SELL).* Similarly, exhausted T-cell levels were calculated based on a negative gene signature (*HAVCR2*, *LAG3*, and *CD279).* The net level of naive and exhausted T cells was calculated by simple subtraction of the enrichment score. This analysis was performed using the contrast gene set function in the Survival Genie 2.0 platform. The Kaplan–Meier curves depict the survival association of naive/exhausted T-cell levels across the following cancers: **(A)** TCGA-BRCA (breast invasive carcinoma), **(B)** TCGA-LUAD (lung adenocarcinoma), **(C)** TCGA-PCPG (paraganglioma and pheochromocytoma), **(D)** TCGA-COAD (colon cancer), **(E)** TCGA-KIRC (kidney renal clear-cell carcinoma), **(F)** TCGA-PAAD (pancreatic adenocarcinoma), **(G)** TCGA-PRAD (prostate adenocarcinoma), and **(H)** TCGA-UVM (uveal melanoma). Each dataset was stratified into high and low groups via the optimal cut-point method (https://pubmed.ncbi.nlm.nih.gov/28642804/) to perform survival association analysis. The association significance was calculated using the log-rank *p*-value.

SUPPLEMENTARY FIGURE S2 Survival analysis hazard ratio plots. K. The analysis provided 95% confidence intervals for the hazard ratio of naive and exhausted T cells. In this analysis, a hazard ratio between 0 and 1 indicated that a higher ratio of naive T cells to exhausted T cells was associated with improved patient survival; a hazard ratio of 1 indicated no effect on survival, and a hazard ratio >1 indicated that a higher ratio of naive T cells to exhausted T cells was associated with worse patient survival.

SUPPLEMENTARY FIGURE S3 Schematic of T-cell antigen labeling and flow cytometry analysis. This diagram details the process of identifying and sorting T cells based on antigen expression using flow cytometry. First, blood is collected to isolate PBMCs, followed by staining with fluorophore-labeled antibodies directed against specific T-cell antigens. This stained cell suspension will be loaded into the flow cytometer for sorting and counting the cells based on fluorescent-labeled antibodies.

SUPPLEMENTARY FIGURE S4 Schematic overview of single-cell RNA sequencing (scRNA-seq) for characterizing immune cells. In scRNA-seq, individual cells are encapsulated into oil-based droplets along with unique barcoded beads, which allow for tagging of transcripts from each cell. The barcoded RNA after library preparation is sequenced and analyzed to quantify the proportions of various cell populations and reveal their transcriptional profiles and functional states, such as naïve T cells, effector T cells, and exhausted T cells.

SUPPLEMENTARY FIGURE S5 Schematic of CyTOF. This diagram details the process of identifying and sorting T cells based on antigen expression using CyTOF in a simple scenario with only one protein of interest. First, PBMCs are collected through a blood sample, and then specific antigens on T cells are labeled with antibodies bonded to heavy metals. This cell suspension is then passed through a mass cytometer, where the isotopes attached to each antibody are detected, allowing for the proportion of tagged (gray) cells and untagged (orange) cells to be analyzed.
