# Supplementary figures and images for: Estimation of biological aging based on T-cell differentiation trajectories: emerging and future avenues
Source: Front Aging. 2025 Nov 20;6:1684051. doi: 10.3389/fragi.2025.1684051 (PMC12675483; doi:10.3389/fragi.2025.1684051)

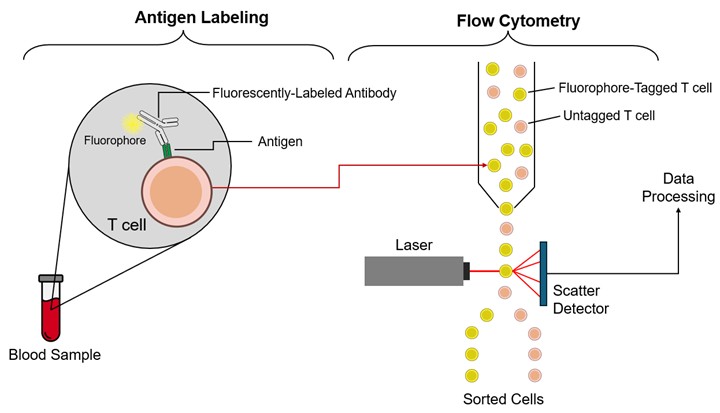

Supplement: Supplementary file 1 [file Image3.jpeg]

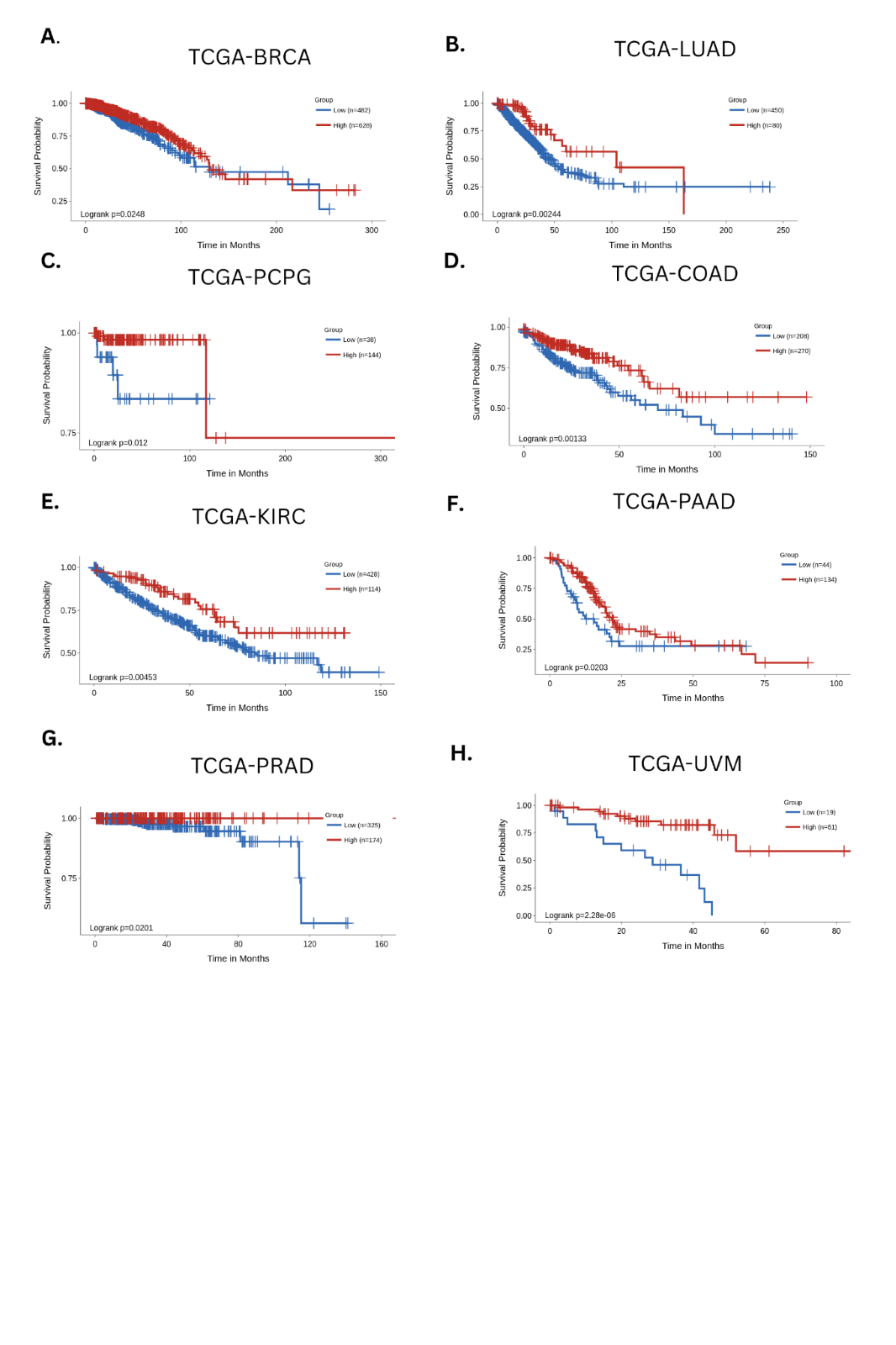

Supplement: Supplementary file 2 [file Image1.jpeg]

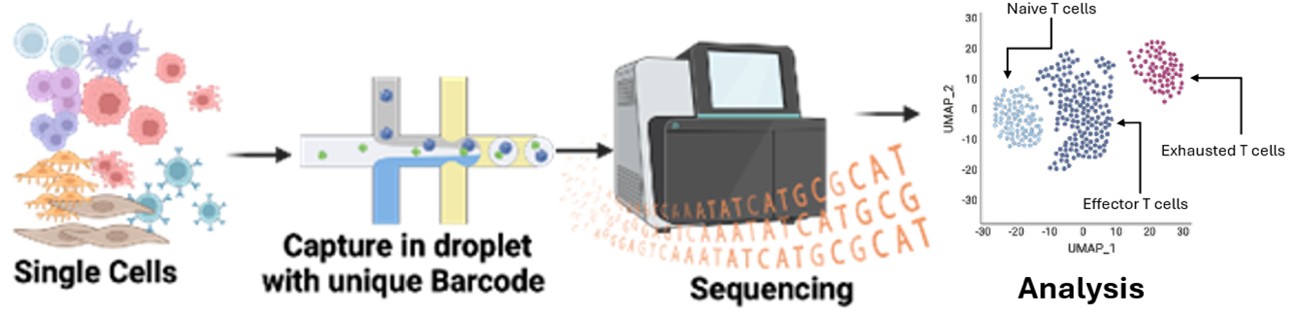

Supplement: Supplementary file 3 [file Image4.jpeg]

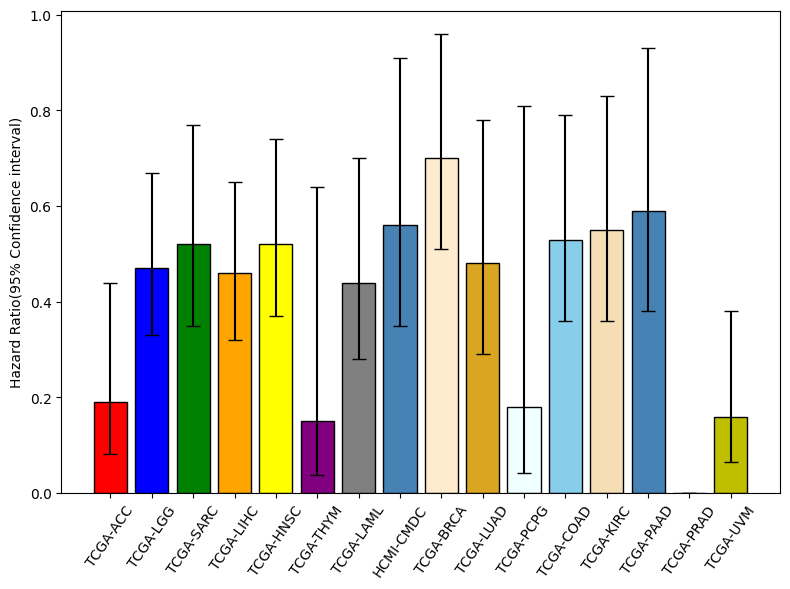

Supplement: Supplementary file 4 [file Image2.jpeg]

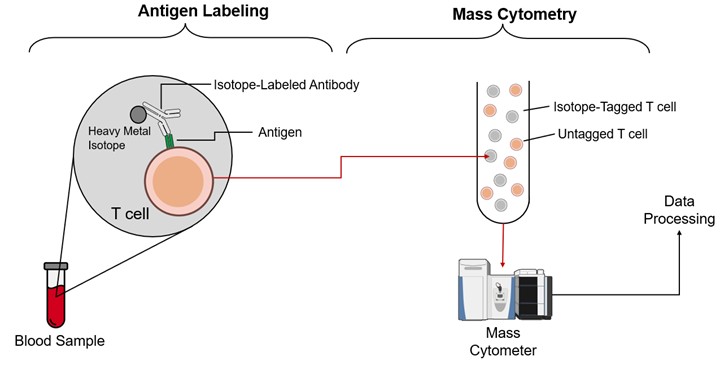

Supplement: Supplementary file 5 [file Image5.jpeg]
